# Foxn1 regulates epidermal structure, redox balance, and age related changes

**DOI:** 10.1186/s12915-026-02737-x

**Published:** 2026-09-16

**Authors:** Sylwia Machcinska-Zielinska, Marta Kopcewicz, Joanna Wisniewska, Karla Valdivieso, Mikolaj Ogrodnik, Katarzyna Walendzik, Barbara Gawronska-Kozak

**Affiliations:** 1 InLife Institute of Animal Reproduction and Food Research, Polish Academy of Sciences, Trylińskiego 18, Olsztyn, 10-683 Poland; 2 Ludwig Boltzmann Institute for Traumatology in AUVA Research Center, Vienna, Austria; 3 Austrian Cluster for Tissue Regeneration, Vienna, Austria

**Keywords:** Skin, Foxn1, Aging, Redox

## Abstract

**Background:**

Skin aging is a complex process, marked by structural decline, functional impairment, and diminished regenerative capacity. In this study, we shine a spotlight on the transcription factor Foxn1 and its pivotal role in modulating age-related changes in skin architecture and redox homeostasis. Using a murine model across three life stages (young — 20-days-old, middle-age — 1-year-old, old — 2-year-old), we compared wild-type (Foxn1^+/+^) and Foxn1 heterozygotic knock out (Foxn1^+/−^) mice to unravel the interplay between genetic regulation and skin aging.

**Results:**

Our findings reveal a striking impact of Foxn1 on skin morphology: Foxn1^+/−^ mice displayed thinner epidermis, thicker dermis, and disrupted keratinocyte differentiation, favoring large spinous layer of epidermal cells. Notably, the skin of Foxn1^+/+^ mice was associated with increased collagen I and a shift toward repair-oriented fibers, whereas Foxn1^+/−^ skin maintained elevated collagen III, hinting at a preserved regenerative profile. Additionally, while p21 expression rose with age in Foxn1^+/+^ mice, it remained low in Foxn1^+/−^ mice, suggesting an altered senescence pathway. Intriguingly, Foxn1 also emerged as a modulator of the hypoxia response, oxidative stress levels, and cellular protection, particularly in young and middle-age mice. These effects diminished with advanced age, underscoring Foxn1’s age-dependent regulatory dynamics.

**Conclusions:**

Together, these results position Foxn1 as one of key regulators of skin homeostasis and reveal its potential role in skin aging processes.

**Supplementary Information:**

The online version contains supplementary material available at https://doi.org/10.1186/s12915-026-02737-x.

## Background

Aging is an inevitable aspect of life, involving structural, biochemical, and functional changes at both the cellular and tissue levels [1]. According to the WHO, the global population aged 60 and older reached 1 billion in 2019 and it is projected to rise to 1.4 billion by 2030 and 2.1 billion by 2050—an unprecedented acceleration that will continue in the coming decades [2].

The skin, the body’s largest organ, is especially vulnerable to aging [3]. Structurally, it consists of three primary layers: the epidermis, dermis, and hypodermis [4]. The epidermis is primarily composed of keratinocytes that produce keratins, forming the protective stratum corneum. The dermis is rich in collagen, elastin, fibronectin, and proteoglycans, which provide tensile strength and mechanical properties. Dermal fibroblasts play a key role in synthesizing and maintaining the collagen-rich extracellular matrix [5].

Skin aging is broadly classified into intrinsic and extrinsic factors affecting aging [6]. Intrinsic aging is driven by physiological changes over time, largely influenced by genetic factors [7]. In contrast, extrinsic aging results from environmental exposures such as ultraviolet (UV) radiation, pollution and lifestyle factors (e.g. nicotine) [8, 9]. Both intrinsic and extrinsic aging contribute to a progressive reduction in the thickness of the epidermis and dermis [10]. In human skin, the number of melanocytes, the pigment producing cells, decreases with age [11]. Age-related structural changes in collagen and elastin [12–14] are directly linked to various skin pathologies, including increased fragility [15], impaired wound healing [16], redox imbalance, hypoxia and oxidative stress [17, 18]. Similar age-related alterations have been observed in murine skin — such as dermal thinning, reduced collagen density, and delayed wound repair [19, 20].

Research on the skin has identified HIF-1α (hypoxia-inducible factor 1-alpha) as a key regulator of skin aging, playing a crucial role in epidermal renewal, wound healing, and oxidative stress regulation. Studies suggest that the decrease of epidermal HIF-1α accelerates aging-related changes, impairing re-epithelialization and contributing to increased oxidative stress [21]. Given that oxidative stress is a major driver of cellular senescence, accumulation of reactive oxygen species (ROS), and extracellular matrix (ECM) degradation, maintaining redox balance is crucial for skin integrity. One of the primary regulators of cellular redox homeostasis is thioredoxin (TXN), a ubiquitous antioxidant protein that declines with age [22]. The decrease in TXN levels exacerbates oxidative damage, promoting cellular senescence and degradation of ECM components such as collagen and elastin [22, 23]. Since HIF-1α and thioredoxin share interconnected roles in cellular responses to oxidative stress, disturbances in their function during aging may collectively contribute to the deterioration of skin structure and function.

In recent years, particular attention has been given to the epidermal transcription factor Foxn1 (Forkhead box N1) as a regulator of skin aging and a modulator of HIF-1α activity and the thioredoxin system [24–28]. Studies using mass spectrometry (LC–MS/MS) and experiments in vitro and in vivo have shown that Foxn1 in keratinocytes regulates elements of the electron transport chain and induces the thioredoxin system (Txn2, Txnrd3, Srxn1), particularly under hypoxic conditions [28]. Moreover, Foxn1 plays a key role in epithelial development, keratinocyte differentiation, skin pigmentation, hair development, and epidermal regeneration [29–31]. In injured skin, Foxn1 is involved in scar-forming healing by influencing re-epithelialization, mechanisms of epithelial-mesenchymal transition (EMT) [24, 32], contributes to the initial stimulation of dermal white adipose tissue (dWAT) adipogenesis and is engaged in the modulation of lipid metabolism processes [33]. Our previous studies have shown that Foxn1 activity gradually declines with age, which may contribute to skin aging process [24, 26]. Furthermore, young and middle-age Foxn1-deficient mice (Foxn1^−/−^), also known as nude mice, exhibit characteristics typical of aged skin, including dryness, wrinkles, epidermal hyperplasia, hair follicle dysgenesis, modified expression of matrix metalloproteinases (MMPs) and their inhibitors (TIMPs) [29, 34].

In the present study, we hypothesized that age-related differences (*young* — 20-days-old*, middle-age* — 1-year-old*,* and *aged* — 2-year-old) and the differences in Foxn1 activity (Foxn1^+/+^ vs. Foxn1^+/−^ mice) alter structure, organization, and redox balance in the skin. To verify this hypothesis, we examined: **(i)** composition, structure and thickness, **(ii)** ECM components, **(iii)** cellular aging markers, and **(iv)** the expression of the redox system components of the skin. The inclusion of Foxn1^+/−^ mice enables us to investigate whether partial inactivation of Foxn1, a gene known to regulate epithelial differentiation and skin homeostasis, is sufficient to influence age-related alterations in skin morphology, redox status, and molecular aging signatures. This model provides a unique opportunity to dissect the gene-dosage-dependent effects of Foxn1 on skin aging and to determine whether a single functional allele can maintain cutaneous homeostasis under physiological aging conditions.

## Results

### The impact of aging and Foxn1 expression on skin structure and thickness

**Fig. 1:**
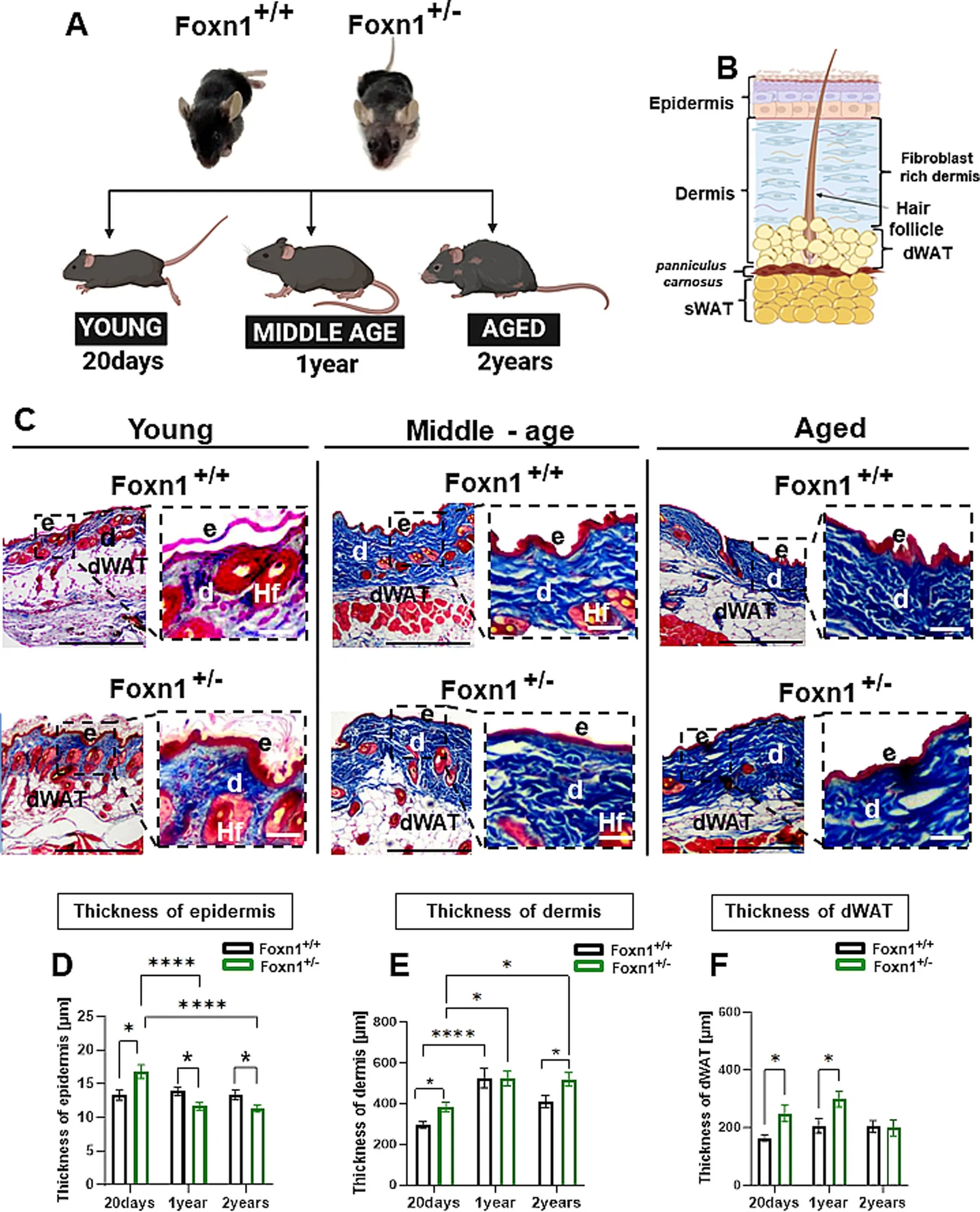
The impact of aging and Foxn1 expression on skin structure and thickness. **A** Schematic representation of the experimental design using young, adult, and old Foxn1^+/+^ and Foxn1^+/−^ mice, **B** scheme of skin structure, **C** histological skin sections stained with Masson trichrome and collected from young (20-days-old), adult (1-year-old) and old (2-year-old) Foxn1^+/+^ and Foxn1^+/−^ mice, quantification of the skin layers thickness: **D** epidermis, **E** dermis and **F** dermal white adipose tissue (dWAT). The measurements of skin thickness were performed on histological slides (*n* = 3–4 per group). e, epidermis; d, dermis; dWAT, dermal white adipose tissue; Hf, hair follicle; scale bar 50 μm, insets 5 μm. The bars indicate mean ± SEM *p < 0.05, ****p < 0.0001. Illustration prepared using BioRender

In this study, we used C57Bl/6 (B6) (Foxn1^+/+^) mice, as well as transgenic Foxn1::eGFP (Foxn1^+/−^) mice, at three age groups: *young* (20-days-old), *middle-age* (1-year-old), and *old* (2-year-old). The transgenic Foxn1::eGFP mice (Foxn1^+/−^) express an eGFP transgene under the control of Foxn1 regulatory sequences (Fig. 1A) [35]. Our previous research established that Foxn1::eGFP mice are Foxn1 heterozygotes (Foxn1^+/−^), where the insertion of the eGFP construct functionally inactivates one endogenous Foxn1 allele [26]. These heterozygous mice exhibit thinning fur and rough, dull hair compared to wild-type (Foxn1^+/+^) mice (Fig. 1A) [26].

To further characterize Foxn1::eGFP mice (Foxn1^+/−^) model at the molecular level, we quantified Foxn1 expression at both the mRNA and protein levels in whole skin lysates from Foxn1⁺^/^⁺ and Foxn1^+/−^ mice at all three studied age points (Additional File 1: Fig. S1). No significant difference in Foxn1 mRNA levels was detected between genotypes at any age point (Additional File 1: Fig. S1A). In contrast, Foxn1 protein levels were significantly elevated in aged Foxn1^+/−^ mice compared to Foxn1⁺^/^⁺ animals at 2 years of age (*p* < 0.01), suggesting post-translational regulation of Foxn1 protein stability in the context of heterozygous eGFP insertion (Additional File 1: Fig. S1B, C; Additional File 2: Fig.S1).

To validate the spatial distribution of Foxn1 within skin compartments, immunofluorescent Foxn1 detection assay was performed on skin sections from Foxn1⁺^/^⁺ and Foxn1⁺^/^⁻ mice across all analyzed age groups (20 days, 1 year, and 2 years). Foxn1 immunoreactivity was identified predominantly in the nuclei of epidermal keratinocytes and cells within hair follicles in both genotypes. A comparable staining pattern was observed across all examined time points, indicating preserved compartment-specific localization of Foxn1 (Additional File 1: Fig.S1D).

Despite these phenotypic observations, the molecular and histological characterization of Foxn1^+/− ^mice across different ages has not yet been performed. To address this gap, we first investigated how changes in Foxn1 expression affect skin morphology by conducting histological analyses to measure the thickness of various skin layers (Fig. 1B, C), including the epidermis (Fig. 1D), dermis (Fig. 1E), and dermal white adipose tissue (dWAT) (Fig. 1F). We also calculated the number of hair follicles (HFs) in the layer of fibroblast rich dermis (Additional File 1: Fig.S2A) and those embedded in dWAT (Additional File 1: Fig.S2B).

The age-related gradual decrease in epidermal thickness was observed exclusively in Foxn1^+/−^ mice, being the thinnest in 2-year-old mice, whereas in Foxn1^+/+^ mice, it remained unchanged over time (Fig. 1D). The differences in epidermal thickness between Foxn1^+/+^ and Foxn1^+/−^ mice were detected at each studied point-age (Fig. 1D). The thickest epidermis was observed in 20-days-old Foxn1^+/−^ mice, while in middle-age and old mice, it was significantly thinner compared to Foxn1^+/+^ mice (Fig. 1D).

The thickness of dermis increased between young and middle-age mice, which was observed for Foxn1^+/+^ and Foxn1^+/−^ mice (Fig. 1E). In both: young and old Foxn1^+/−^ mice, the dermis was significantly thicker than in Foxn1^+/+^ mice, while for middle-age there were no significant differences (Fig. 1E).

The thickness of dWAT was stable across aging for: Foxn1^+/+^ and Foxn1^+/−^ mice (Fig. 1F). However, dWAT was significantly thicker in young and middle-age Foxn1^+/−^ compared to Foxn1^+/+^ mice at the same age (Fig. 1F). The number of HFs in the dermis showed no significant differences between Foxn1^+/−^ and Foxn1^+/+^ mice at each age studied timepoint (Additional File 1: Fig.S2A). The age-related decrease in the number of hair follicles in dWAT was detected in both Foxn1^+/−^ and Foxn1^+/+^ mice, with the lowest number observed in old Foxn1^+/−^ mice compared to Foxn1^+/+^ mice (*p* < 0.05; Additional File 1: Fig. S2B).

### Partial inactivation of Foxn1 (Foxn1^+/-^ mice) affects the composition of epidermis

**Fig. 2:**
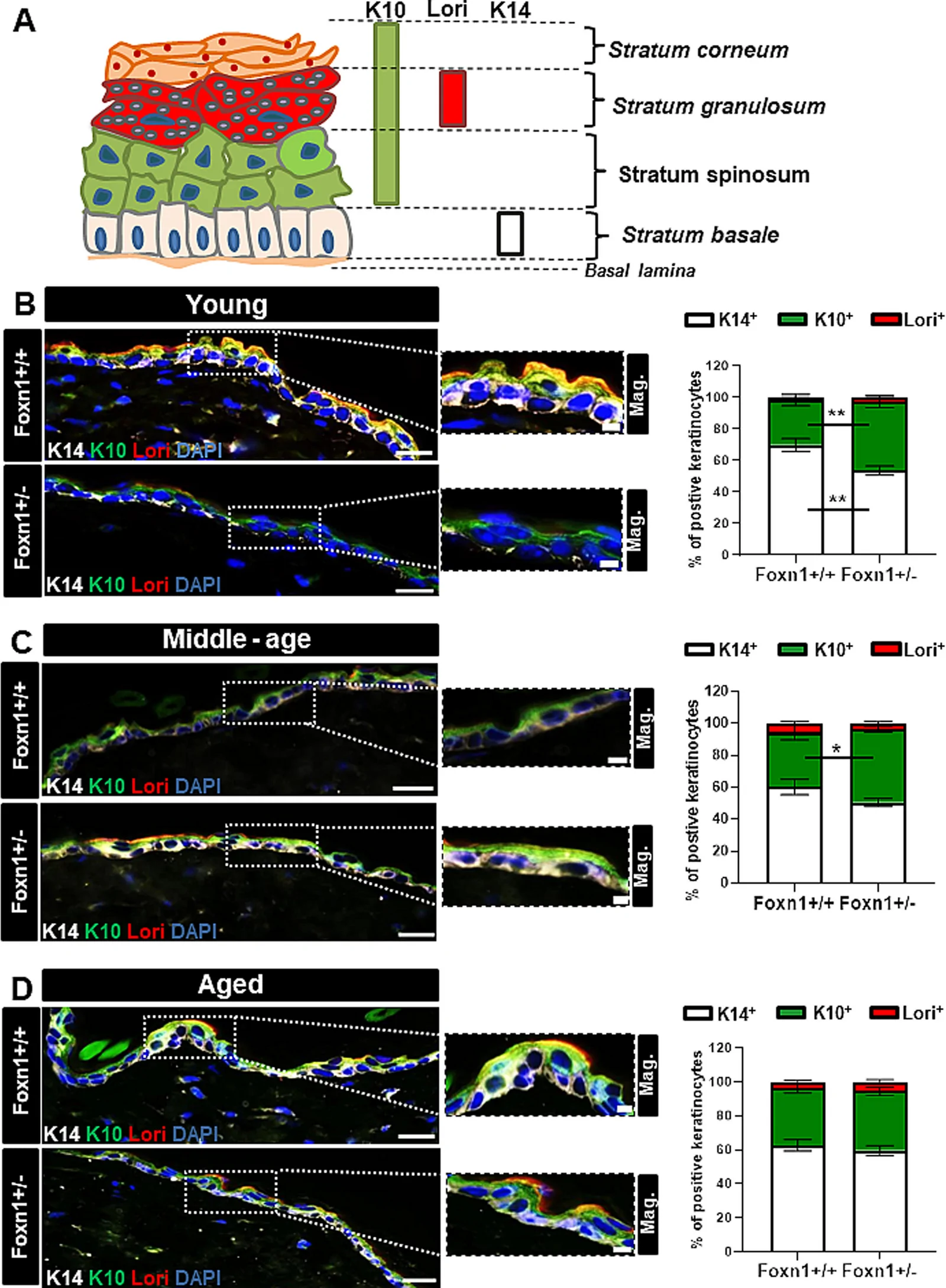
Partial inactivation of Foxn1 (Foxn1^+/− ^mice) affects composition of epidermis.** A** Schematic representation of the skin showing differentiated keratinocytes in the epidermis and expression of stratification markers: Keratin 10 (K10), Keratin 14 (K14), and Loricrin (Lori). **B**–**D** immunofluorescence detection of K10, K14, and Lori, along with quantification of the percentage of positive keratinocytes in each epidermal layer in young (20-day-old), adult (1-year-old), and old (2-year-old) Foxn1^+/+^ and Foxn1^+/−^ mice (*n* = 3–4 per group). Scale bar: 20 μm; insets: 5 μm. The bars indicate mean ± SEM *p < 0.05, **p < 0.01

To evaluate the effect of aging and partial inactivation of Foxn1 on epidermis, immunofluorescent staining was performed for: keratin 14 (K14; predominantly expressed in the *stratum basale*), keratin 10 (K10; localized to suprabasal cells; *stratum spinosum*) and loricrin (Lori; characterized for *stratum granulosum* as the outermost layer of epidermis) (Fig. 2A–D).

The higher percentage of K14^+^ keratinocytes was detected in young (20-days-old; *p* < 0.01) and middle-age (1-year-old; non-statistical) Foxn1^+/+^ compared to Foxn1^+/−^ mice (Fig. 2B, C). However, the percentage of K10⁺ keratinocytes was lower in young Foxn1^+/+^ than in young Foxn1⁺^/^⁻ mice. No significant differences were observed in the percentage of Lori^+^ keratinocytes between Foxn1^+/+^ and Foxn1^+/−^ (Fig. 2B, C, D). In aged animals, no significant differences in the percentage of K14^+^, K10^+^ or Lori^+^ cells between Foxn1^+/+^ and Foxn1^+/−^ mice were detected (Fig. 2D).

### Age- and Foxn1-dependent alterations modify expression and structure deposition of collagen

**Fig. 3:**
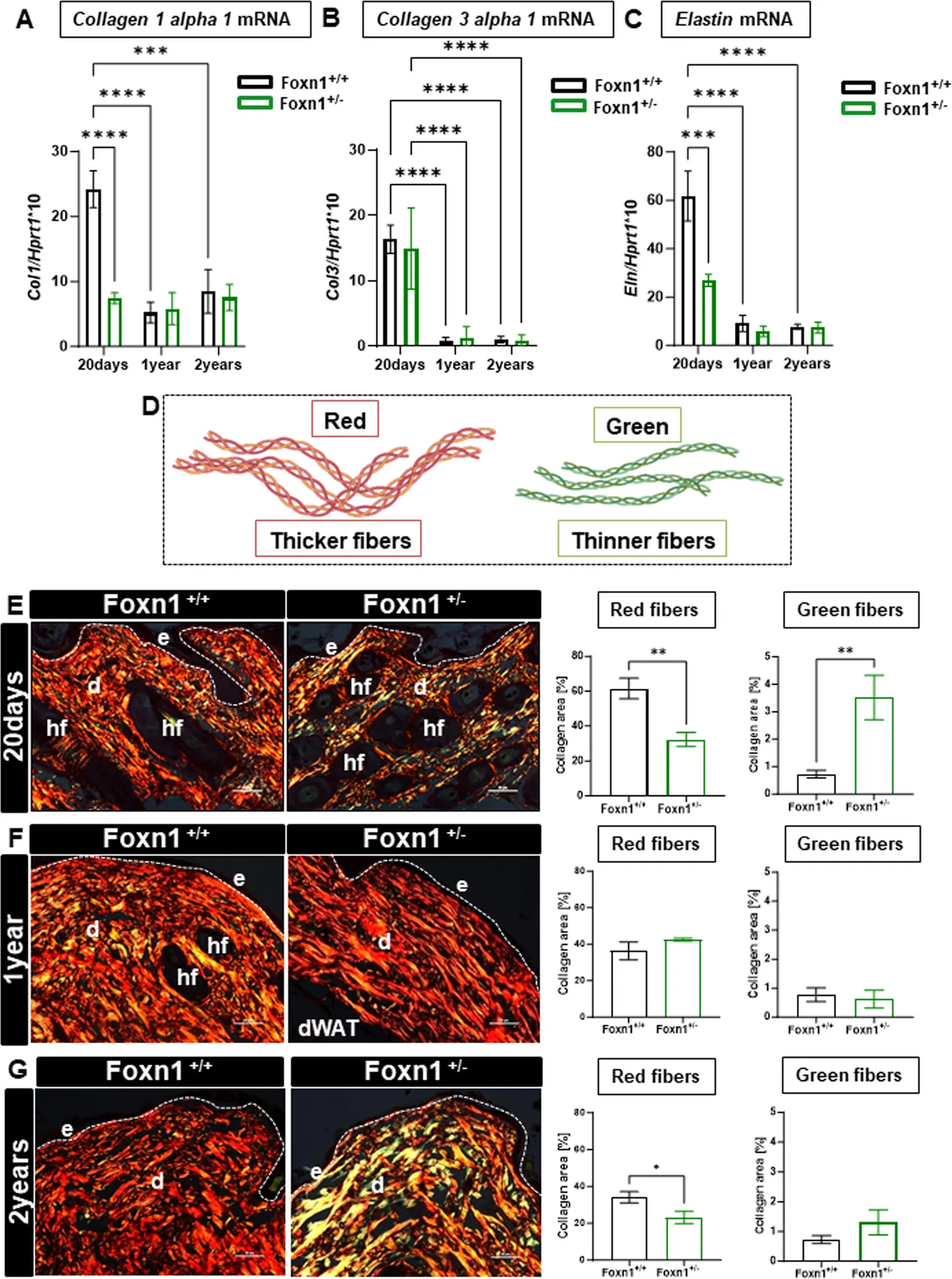
Age- and Foxn1-dependent alterations modify collagen skin expression and deposition.** A** Collagen 1, **B** Collagen 3, and **C** Elastin mRNA expression in skin of young (20-day-old), adult (1-year-old), and old (2-year-old) Foxn1^+/+^ and Foxn1^+/−^ mice (*n* = 8 skin samples per group), **D** Collagen fiber diagram, **E**–**G** Evaluation of collagen type I (red fibres) vs. collagen type III (green fibres) content in stained picrosirius red histological skin sections collected from young, adult and old Foxn1^+/+^ and Foxn1^+/−^ mice (*n* = 3–4 per group). e, epidermis; d, dermis; dWAT, dermal white adipose tissue; Hf, hair follicle; scale bar 20 μm. The bars indicate mean ± SEM *p < 0.05, **p < 0.01

To quantitatively analyze the impact of age and Foxn1 level on structure of dermis, the mRNA expression levels of the ECM components: collagen type I (*collagen 1 alpha 1*), collagen type III (*collagen 3 alpha 1*) and *elastin* (Fig. 3A–C) were measured. Quantitative real time PCR (RT-qPCR) analysis revealed that the skin of 20-days-old Foxn1^+/+^ mice expressed the highest levels of *collagen 1* (Fig. 3A). In contrast, the skin of 20-days-old Foxn1^+/−^ mice displayed low *collagen I* expression (*p* < 0.001) that was constant among Foxn1^+/−^ age-groups (Fig. 3A). *Collagen III* expression was similar between Foxn1^+/+^ and Foxn1^+/−^ regardless of age, being the highest at 20-days-old mice followed by a gradual decline with age (Fig. 3B). The expression of *elastin* followed the pattern observed for *collagen I* that was significantly higher in the skin of young Foxn1^+/+^ mice compared to Foxn1^+/−^, and then the levels declined with age (Fig. 3C). To further characterize the collagen content, histological sections of the skin stained with picrosirius red were examined under polarized light to distinguish collagen fibers: type I (red, thicker fibers) from type III (green, thinner fibers) [36] (Fig. 3D, E). We followed methodology developed by *Wolf *et al*.* [37] to ensure consistent qualitative and quantitative assessment of ECM collagen alterations across all specimens. As shown in Fig. 3E–G, the section background appears black, and the collagen bundles are red (the thickest), orange/yellow (medium thickness, transitional) and green (the thinnest). The highest content of red fibers was detected in young Foxn1^+/+^mice (61.52%) in comparison to the young Foxn1^+/−^ mice (32.28%) (Fig. 3E). The area occupied by transitional collagen fibers (orange and yellow) was higher in young Foxn1^+/−^ mice (55.91% and 3.51%, respectively) than in Foxn1^+/+^ mice (34.86% and 2.88%) (Additional File 1: Fig.S3A, B). The level of thin green fibers was significantly higher in the skin of young Foxn1^+/−^ mice (3.51%) compared to young Foxn1^+/+^ mice (0.72%) (Fig. 3E). The skin of middle-age mice showed similar level of thick and thin fibers between Foxn1^+/+^ and Foxn1^+/−^ mice (Fig. 3F). In middle-age Foxn1^+/+^ and Foxn1^+/−^ skin, red and transitional (orange/yellow) fibers dominated, while green fibers accounted for less than 1% (Fig. 3F, Additional File 1: Fig.S3C–D). Old Foxn1^+/+^ mice showed higher red fiber content than Foxn1^+/−^ (34.11% vs. 23.28%) (Fig. 3G). Orange fibers were similar between groups (≈53–56%), while yellow fibers were more abundant in Foxn1^+/−^ (19.52% vs. 12.30%) (Additional File 1: Fig.S3E, F). The proportion of green fiber levels were low and not significantly different (1.31% vs. 0.73%) (Fig. 3G).

### Partial inactivation of Foxn1 in the skin alters the expression of aging markers in old mice

**Fig. 4:**
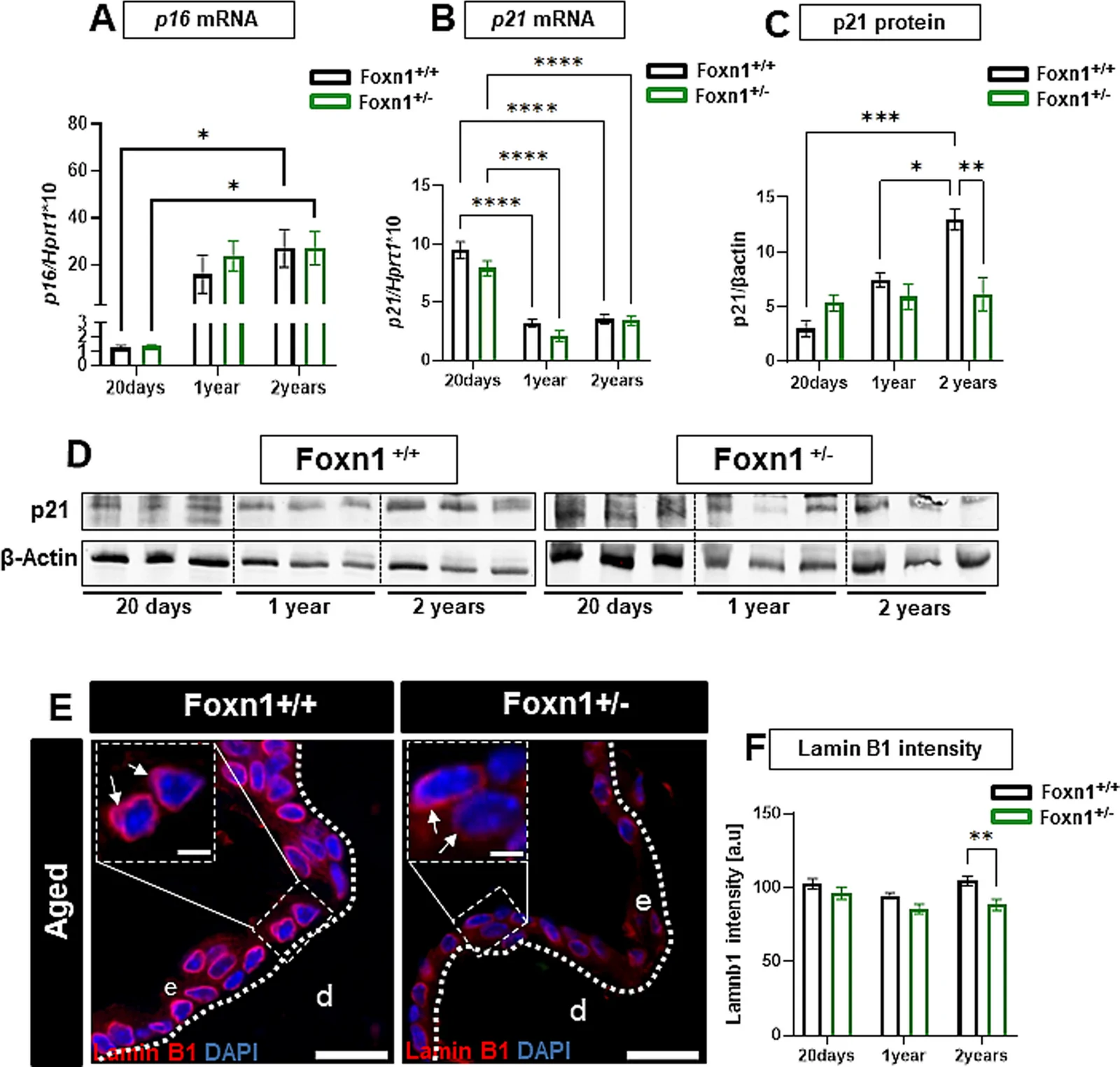
Partial inactivation of Foxn1 in the skin (Foxn1^+/−^ mice) alters the expression of aging markers in old mice. **A** *p16*, **B** *p21* mRNA expression in skin of young (20-day-old), adult (1-year-old), and old (2-year-old) Foxn1^+/+^ and Foxn1^+/−^ mice (*n* = 8 skin samples per group), **C**, **D** densitometric analysis and representative Western blot of p21 and β-actin (reference protein) in skin collected from young, adult, and old Foxn1^+/+^ and Foxn1^+/−^ mice (*n* = 3 skin samples per group). **E** Immunofluorescent detection of Laminb1 in epidermal layer of the skin collected from old Foxn1^+/+^ and Foxn1^+/−^ mice, **F** calculation of Laminb1 intensity signal in epidermal layer of the skin collected from young, adult, and old Foxn1^+/+^ and Foxn1^+/−^ mice (*n* = 3–4 per group). Dotted lines separate epidermis from dermis. Arrows depict Laminb1- positive cellse—epidermis, d- dermis. Scale bar: 20 μm; insets: 5 μm. The bars indicate mean ± SEM *p < 0.05, **p < 0.01, ***p < 0.001, ****p < 0.0001

To assess the impact of aging on the skin, senescence markers between Foxn1^+/+^ and Foxn1^+/−^ mice the expression of *p16* and *p21* mRNA (Fig. 4A, B), protein levels of p21 (Fig. 4C, D), and the intensity of Lamin B1 (Fig. 4E, F) were analyzed. RT-qPCR analysis revealed that *p16* mRNA skin expression levels increased with the course of aging of both Foxn1^+/+^ and Foxn1^+/−^ mice (Fig. 4A), whereas *p21* levels of mRNA expression declined over time (Fig. 4B). Next, p21 protein levels in the skin of Foxn1^+/+^ and Foxn1^+/−^ mice were examined using western blot (Fig. 4C, D; Additional File 2: Fig. S2). In the skin of Foxn1^+/+^ mice, a progressive age-related increase in p21 protein levels was observed, with the highest peak detected in old Foxn1^+/+^ mice (*p* < 0.01, Fig. 4C, D: Additional File 2: Fig. S2). In contrast, Foxn1^+/−^ mice exhibited low p21 protein levels that were not affected by age (Fig. 4C: Additional File 2: Fig. S2).

Immunofluorescent detection demonstrated the presence of Lamin B1-positive cells surrounding the nuclear envelope in keratinocytes (Fig. 4E). The intensity of Lamin B1 staining in the skin of 20-day-old and 1-year-old of both: Foxn1^+/+^ and Foxn1^+/−^ mice was comparable (Fig. 4E, F). A significant loss of Lamin B1 was detected in aged Foxn1^+/−^ keratinocytes compared to Foxn1^+/+^ mice (Fig. 4E, F).

### Age and partial depletion of Foxn1 alter skin redox balance

**Fig. 5:**
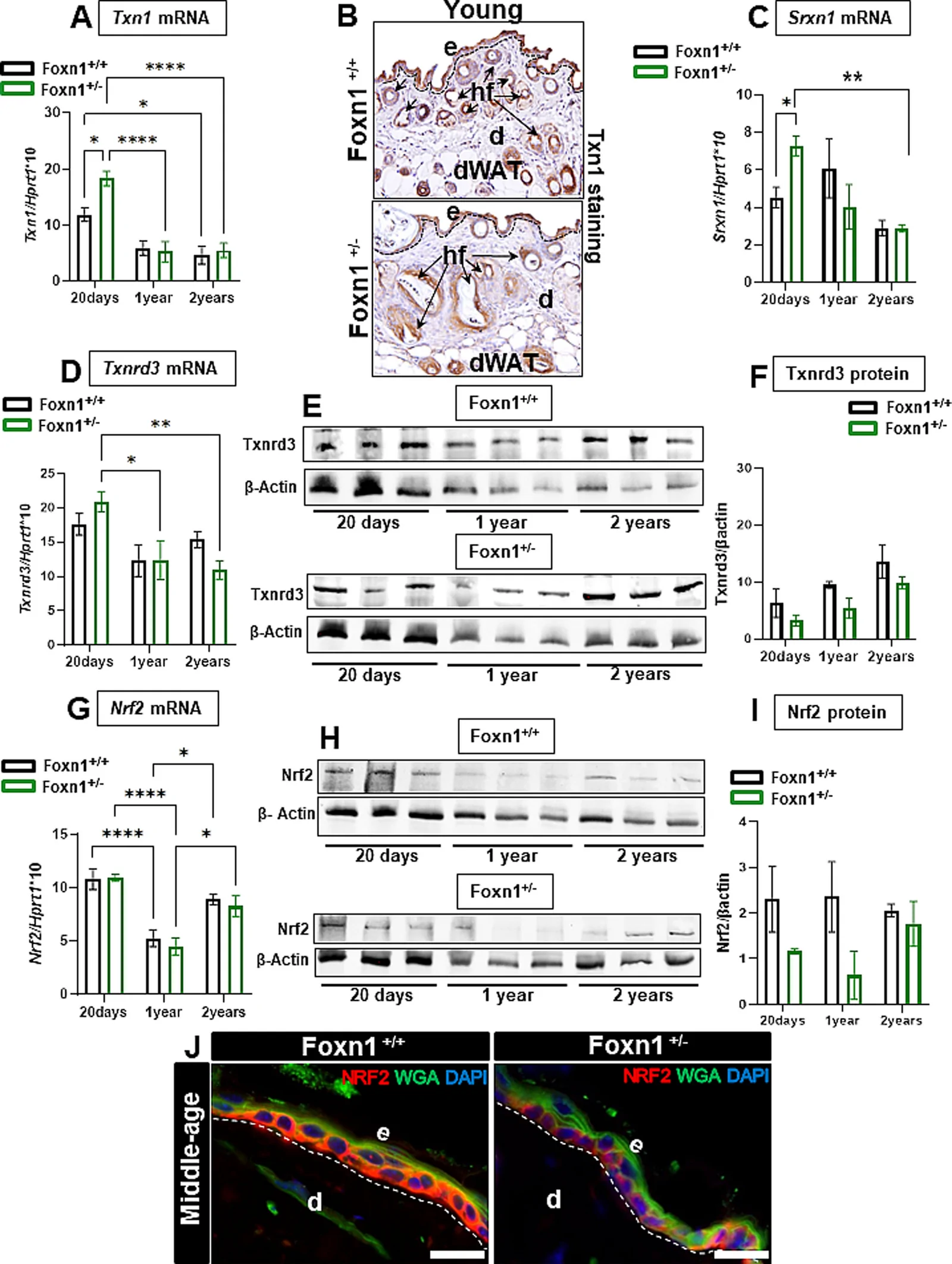
Age and Foxn1 expression (Foxn1^+/+^ vs Foxn1^+/−^ mice model) alters skin redox balance.** A** Txn1, **C** Srxn1, **D** Txnrd3 and **G** Nrf2 mRNA expression in skin of young (20-day-old), adult (1-year-old), and old (2-year-old) Foxn1^+/+^ and Foxn1⁺^/^⁻ mice (*n* = 8 skin samples per group), **E**, **F** Txnrd3 and **H**, **I** Nrf2 densitometric analysis and representative Western blot with β-actin (reference protein) in the skin of young, adult, and old Foxn1^+/+^ and Foxn1^+/−^ mice (*n* = 3 skin samples per group). **B** Immunohistochemical detection of Txn1 in 20-days-old skin of Foxn1^+/+^ and Foxn1^+/−^ mice. **J** Immunofluorescent detection of Txnrd3 and Wheat Germ Agglutinin (WGA) — used for plasma membrane labeling and general cel vizualization) in 1-year-old skin of Foxn1^+/+^ and Foxn1^+/−^ mice. e, epidermis; d, dermis; dWAT, dermal white adipose tissue; Hf, hair follicle; scale bar **B** 200 μm, **J** 20 μm. The bars indicate mean ± SEM *p < 0.05, **p < 0.01 ****p < 0.0001

Given our previous findings that Foxn1 regulates the thioredoxin system [27, 28], we analyzed redox-related factors, including Txn1, Txn2, Txnrd1–3, Srxn1, and Nrf2, in the skin of Foxn1^+/+^ and Foxn1^+/−^ mice during aging (Fig. 5). Txn1 expression was highest in young mice regardless of genotype, with significantly elevated levels in Foxn1^+/−^ compared to Foxn1^+/+^ (Fig. 5A). Immunohistochemistry confirmed Txn1 localization in the epidermis and hair follicles of both genotypes (Fig. 5B, Additional File 1: Fig. S4A). No significant differences were observed for Txn2, Txnrd1 or Txnrd2 (Additional File 1: Fig. S4B, D and S4C). Srxn1 followed a similar pattern to Txn1, with highest expression in young mice, especially in Foxn1^+/−^ skin, and a decline with age (Fig. 5C). Txnrd3 mRNA showed age-related decline in Foxn1^+/−^ mice (Fig. 5D), while its protein levels increased with age in both genotypes (Fig. 5E, F; Additional File 2: Fig. S3). Confocal microscopy using Foxn1::eGFP skin confirmed colocalization of Foxn1 and Txnrd3 in basal keratinocytes (Additional File 1: Fig. S4E). Nrf2 mRNA showed the highest expression in young skin, a drop in middle age, and a rebound in aged mice (Fig. 5G), while protein levels were consistently higher in Foxn1^+/+^ mice, though not statistically significant (Fig. 5H, I: Additional File 2: Fig. S3). Immunofluorescence revealed strong Nrf2 signal in 1-year-old Foxn1^+/+^ epidermis, with weak signal in Foxn1^+/−^ (Fig. 5J). Finally, analysis of Hif-1α mRNA levels showed no differences (Additional File 1: Fig. S5A), but nuclear protein levels were higher in young and middle-aged Foxn1^+/+^ mice (Additional File 1: Fig. S5B, C: Additional File 2: Fig. S4). Hif-1α was undetectable in aged mice, and Fih-1 expression remained unchanged across age and genotype (Additional File 1: Fig. S5D–F: Additional File 2: Fig. S4).

## Discussion

This study evaluated the impact of aging and Foxn1 levels on skin structure, differentiation processes in epidermis, and redox balance. The results indicate that partial loss of Foxn1 correlates with an age-dependent decrease in epidermal thickness, a simultaneous increase in dermal thickness, and alterations in the expression of keratinocyte differentiation markers. Furthermore, Foxn1⁺^/^⁻ mice exhibited lower levels of thick and a higher proportion of thin collagen fibres. Differences in p21 protein levels were also observed, along with an impact of Foxn1 on hypoxia regulation, oxidative stress, and cytoprotective mechanisms, particularly in young and middle-age skin, with these effects diminishing in aged tissue.

Foxn1 is a key regulator of keratinocyte differentiation and mutations in the Foxn1 gene lead to severe defects in keratinization and hair follicle structure [30]. Dysfunctional differentiation of keratinocytes is also observed in aging of human skin displayed as progressive epidermal thinning [38]. While mice are infrequently reported to show age-related epidermal thinning, we observed that Foxn1^+/−^ mice exhibited a marked reduction in thickness of epidermis at 1 and 2 years old. It is thus exciting to speculate that Foxn1^+/−^ mice show human-like features of skin aging. Consistent with the importance of Foxn1 in keratinocytes, these aging-like features in Foxn1^+/−^ mice are primarily restricted to the epidermis, as we did not observe thinning of the dermal or adipose compartments, changes that are known to occur during human skin aging [38].

Collagens particularly types I and III play a central role in maintaining the skin’s structural integrity, mechanical strength, and elasticity. Its synthesis decreases with age, leading to weakened skin and increased vulnerability to damage [39, 40]. Our study corroborates this statement, showing a significant reduction in mRNA expression of *collagen I* and *elastin* in aged Foxn1^+/+^ mice. In contrast, in Foxn1^+/−^ mice, the levels of *collagen I* and *elastin* remained constantly low regardless age (young, middle-age or old). Histological analysis revealed that the dermal part of the skin of Foxn1^+/+^ mice exhibited a dominance of type I collagen fibers, whereas Foxn1^+/−^ mice predominantly showed type III collagen. Given that type III collagen is associated with scar-free healing ability of Foxn1^−/−^ mice [34], the results suggest that partial loss of Foxn1 (Foxn1^+/−^) promotes a regenerative healing profile by increasing collagen III relative to collagen I.

It has been demonstrated that the progressive decline in the skin’s antioxidant activity with age leads to an increase in reactive oxygen species (ROS), inducing oxidative stress responsible for premature skin aging [41]. Redox balance analysis revealed that components of the thioredoxin system (Txn1 that expressed in cytoplasma and Srxn1) exhibited the highest expression levels in young skin (20-days-old), particularly in Foxn1^+/−^ mice that gradually declined with age. These findings support previous evidence indicating the crucial role of thioredoxin in cytoprotection during early developmental stages [42, 43]. Notably, the colocalization of Foxn1 and Txnrd3 in basal keratinocytes is consistent with its proposed role in epidermal homeostasis and repair [27], further confirming the significance of Foxn1 in redox regulation.

Another crucial aspect of the present study is the role of Foxn1 in skin aging. While most research has focused on thymic tissues, where Foxn1 mRNA expression decreases with age [44–47], only a few studies have explored the loss of Foxn1 in skin during aging [26, 48]. Our data show that Cdkn2a (p16) mRNA expression increased with age in both Foxn1^+/+^ and Foxn1^+/−^ mice, consistent with its role as a well-established marker of cellular senescence [49, 50]. Yet, there were no differences between the genotypes observed. However, while the skin of Foxn1^+/+^ mice exhibited an age-related increase in p21 protein levels, Foxn1^+/−^ mice displayed a reduced p21 levels across all age groups. Considering an important role of p21 in keratinocyte differentiation [51], a low level of this protein could be in this context more directly related to impaired keratinization of the Foxn1-deficient mice rather than a feature of senescence. While reduced levels of Lamin B1 in Foxn1^+/−^ mice could indicate a higher level of senescence [52], which is known to occur during both aging and healing, a single marker is insufficient to reliably confirm the presence of senescent cells [50]. Therefore, more research is needed to clarify the relationship between senescence and Foxn1 deficiency. Given the pronounced phenotype of age-dependent epidermal thinning, it is intriguing to speculate that other features associated with skin aging, such as stem cell depletion, may also be involved. This will be the subject of our future study.

Foxn1 is expressed not only in the skin but also in the thymic epithelium, where it plays a fundamental role in thymic epithelial cell differentiation and T cell development [53]. Although the present study focused exclusively on the cutaneous consequences of Foxn1 haploinsufficiency, altered thymic Foxn1 activity could indirectly influence skin homeostasis through immune-mediated mechanisms. As no thymic tissue or immune parameters were analyzed, a contribution of thymic alterations to the observed skin phenotype cannot be excluded. Future studies employing tissue-specific Foxn1 loss-of-function models together with comprehensive immunological profiling will be required to distinguish direct cutaneous effects from potential thymus-mediated mechanisms.

More broadly, skin aging is increasingly recognized as a multifactorial process driven not only by intrinsic cellular aging but also by chronic low-grade inflammation (inflammaging), impaired epidermal barrier function, and dysregulated immune responses [54]. Within this framework, altered Foxn1 activity may affect epidermal homeostasis while interacting with inflammatory pathways that contribute to age-associated skin remodeling.

## Study limitations

The present study has several limitations. First, Foxn1 expression was assessed in whole skin lysates obtained from germline Foxn1^+/−^ mice rather than in individual skin cell populations. Consequently, the present study does not allow discrimination between cell-autonomous effects within keratinocytes and indirect systemic effects resulting from Foxn1 haploinsufficiency. Although publicly available single-cell RNA sequencing datasets (DigiSkin Atlas) [55] indicate that Foxn1 expression is largely restricted to keratinocytes, future studies employing cell type-resolved analyses together with keratinocyte-specific conditional Foxn1 deletion models will be required to define the skin-intrinsic functions of Foxn1 during aging.

Second, the Foxn1::eGFP model combines Foxn1 haploinsufficiency with expression of an eGFP-containing construct under the control of Foxn1 regulatory sequences. Although this model has been previously validated and the observed phenotype is consistent with reduced functional Foxn1 dosage, we cannot formally exclude a contribution of the reporter construct itself to the phenotype. Future studies comparing this model with independent Foxn1 loss-of-function models, such as mice carrying a heterozygous Cre-mediated Foxn1 deletion without a reporter cassette, would further strengthen the mechanistic interpretation of the observed phenotype.

Finally, functional analyses of the skin — including barrier function, wound healing capacity, and tensile strength — were not performed in the present study. The impact of partial Foxn1 deficiency on these functional properties of the skin remains an important open question that should be addressed in future studies.

## Conclusion

Collectively, our data are the first to demonstrate that partial loss of Foxn1 (Foxn1^+/−^) impacts skin structure and molecular profile with certain features resemblant of those observed during aging. Future studies aiming to investigate the exact mechanism of the Foxn1-dependent skin aging process and its regulatory pathway in the skin are necessary.

## Methods

### Animals

For this study, we used samples of intact skin of young (20-days-old, total *n* = 8), middle-age (1-year-old, total *n* = 8), and old (2-year-old, total *n* = 8 B6 wild-type (C57BL/6; Foxn1^+/+^) mice, as well as genetically matched heterozygous transgenic Foxn1::Egfp (Foxn1^+/−^) mice, in which the expression of eGFP is driven by Foxn1 regulatory sequences [24, 26, 32, 35]. The Foxn1^+/−^ mice were generously provided by Professor Thomas Boehm (Max Planck Institute of Immunobiology and Epigenetics, Germany). The age categories used throughout this study follow the convention commonly adopted in laboratory mouse aging research, in which mice aged approximately 10–15 months are considered middle-aged and those aged 18–24 months are considered old [56]. Furthermore in the present study we followed established in our laboratory model of old 18–20 month old B6 mouse model [24, 57]. All animals were bred and housed under standard conditions in a temperature (22 ± 2 °C) and humidity-controlled (35–65%) environment with a 12:12 h light/dark cycle at the Institute of Animal Reproduction and Food Research, Polish Academy of Sciences, Olsztyn, Poland. Animals were euthanized by exposure to an overdose of carbon dioxide (CO₂), followed by cervical dislocation to ensure death, after which dorsal skin samples were collected post-mortem. No experimental procedures were performed on live animals in the present study. Ethical approval was therefore not required, as the animals were euthanized solely for the collection of tissues for post-mortem analyses. Animal handling and euthanasia were performed in accordance with applicable national regulations and Directive 2010/63/EU on the protection of animals used for scientific purposes.

### Histology

Formalin-fixed skin samples (*n* = 3–4 per group) were processed, embedded in paraffin, and sectioned at 5 µm.

The thickness of the skin layers was assessed on skin samples stained with a Trichrome Stain Kit (Trichrome Stain Kit, Abcam) according to the manufacturer’s instructions. On each histological slide (*n* = 3–4 per animal group), three separate, non-overlapping measurements were taken for each skin layer and used for subsequent analysis. The thickness of individual skin layers and the number of hair follicles were quantified using ImageJ software (ImageJ, National Institutes of Health, USA).

Slides for highlight collagen networks (collagen type I and collagen type III) were stained with picrosirius red (Direct Red 80, Catalog# 36–554–8, Sigma-Aldrich Co.) for 1 h. After staining, sections were washed in two changes of acidified water (0.5% acetic acid) and dehydrated with three changes of 100% ethanol. The sections were visualized using Nikon eclipse Ci with Stereo Microscope Polarizer Filter Lens for SMZ-U Base. The area of collagen fibers as a function of their color hue was quantified from tissue sections stained with picrosirius red and imaged with circularly polarized light microscopy (200 × magnification). The color hue corresponds to relative fiber thickness from thin green fibers to increasingly thick yellow, orange, and red fibers as collagen content for each color component as a percentage of the area of each image (previously described by *Wolf*) [37].

Immunohistochemical staining for the presence of Txn-1 (1:200, Cat# 24,295, Cell Signaling) was performed on consecutive skin sections. Antibody binding was detected with the ABC complex (Vectastain ABC kit, Vector Laboratories, Inc). In the control sections, primary antibodies were substituted with nonspecific immunoglobulin G (IgG). Peroxidase activity was revealed using 3,3′-diaminobenzidine (Sigma-Aldrich) as a substrate. The slides were counterstained with hematoxylin (Sigma-Aldrich). The sections were visualized using an Olympus microscope (BX43), photographed with an Olympus digital camera (XC50), and analyzed with Olympus CellSens Software.

Immunofluorescence detection of Foxn1 (1:100, Cat#ab113235, Abcam), Keratin 10 (K10) (1:400, Cat#GP-K10, Progen), Keratin 14 (K14) (1:200, Cat#ab7800, Abcam), Loricrin (Lori) (1:250, Cat#ab198994, Abcam), LaminB1 (1:400, Cat# ab16048, Abcam), Nrf2 (1:100, Cat#PA5-67,520, ThermoFisher), WGA dye (20 min at 37°; 1:200, Cat#W11261, ThermoFisher) was performed on skin sections. Tissue sections underwent deparaffinization and rehydration through a graded alcohol series. To retrieve antigens through heat induction, samples were incubated in a steamer at 95 °C for 20 min using a 0.1 M Tris with 0.01 M EDTA (pH 9.0) or sodium-citrate buffer 0.01 M (pH 6.0), or in the microwave where the buffer was brought to a boil and then left for 15 min at sub-boiling temperature.

Samples were blocked in PBS containing 0.4% Bovine serum albumin (BSA, Sigma Aldrich) and 1.6% normal goat serum (NGS, Vectorlabs) for 1 h at room temperature. Subsequently, samples were incubated with appropriate primary antibodies in blocking buffer overnight at 4 °C. Slides were washed three times with TBS-Tween (TBS-T) and incubated with fluorescent secondary antibody Alexa Fluor 488 (1:1000, Cat# A-11073, Invitrogen), Alexa Fluor 647 (1:1000, Cat# A-11032, Invitrogen) and Alexa Fluor 594 (1:1000, Cat# A-11037) for 1 h at room temperature. Afterward, selected sections were stained with WGA dye for 15 min then all sections applied DAPI (1:1000; HY-D0814, MCE) for 15 min and mounted using MOWIOL mounting medium (Carl Roth). Keratinocyte differentiation was assessed using the markers K10, K14, and Lori, and intensity of borders surrounding keratinocytes was measured using marker Lamin B1. Epidermal cells were analyzed from four 40 × images using inverted microscope Nikon Eclipse Ni (Nikon Corporation, Tokyo, Japan), quantifying the total number of cells and measuring individual cell sizes across the different strata. Planimetric analysis was conducted using Fiji image processing software (ImageJ, National Institutes of Health, USA) to perform these measurements.

Immunofluorescence of Foxn1 (eGFP) (1:300, Cat # ab6673, Abcam), and Txnrd3 (1:300, Cat# 19,517–1-AP, ThermoFisher) were performed on skin samples from young (20-days-old) Foxn1::Egfp transgenic mice. The samples were fixed in 4% PFA for 2 h, washed in 0.1 M phosphate buffer (PB) (Sigma-Aldrich) overnight at 4 °C and stored in 18% sucrose (Chempur, Poland) prior to cryosectioning. Immunofluorescence assays were performed with the following primary antibodies: anti-Trx-1 (1:50, Cat# 24,295, Cell Signaling) and anti-eGFP (1:400, Cat# 6673, Abcam). Alexa Fluor 594 (1:200, Cat# A11037, Invitrogen) and Alexa Fluor 488 (1:200, Cat# A 21206, Invitrogen) secondary antibodies were used. Nuclei were counterstained with ProLong Gold Antifade Mountant with DAPI (Invitrogen). The sections were visualized and photographed with an Olympus microscope (BX43) equipped with an Olympus digital camera (XC50) and analyzed with CellSens Dimension Software (Olympus Soft Imaging Solutions GmbH). Confocal images were scanned and digitalized using an F10i Laser Scanning Microscope integrated with Fluoview Software (Olympus) with a 10 × and 60 × objective lens. The sequential scans were acquired with Z spacing of 0.5 μm and 512 × 512 pixel size.

### Protein isolation and Western blot analysis

Frozen skin samples (*n* = 3 per group) collected from young (20-days-old), middle-age (1-year-old) and old (2-year-old) Foxn1^+/+^ and Foxn1^+/−^ mice were powdered in liquid nitrogen using a prechilled mortar and pestle, and then, homogenized in RIPA buffer containing protease inhibitor cocktail (Sigma-Aldrich), phosphatase inhibitor cocktail (Sigma-Aldrich), and phenylmethanesulfonyl fluoride (PMSF, Sigma-Aldrich), followed by sonication (3 × 5 s) with a sonicator (Vibro-Cell VCX 130 PB). For HIF-1α, Fih-1 protein levels estimation the nuclear fraction of homogenates were lysed in buffer (NE-PER; Nuclear and Cytoplasmic Extraction Reagents; ThermoFisher Scientific, Rockford, USA) supplemented with protease inhibitor cocktail. Protein concentration was measured by the infrared (IR)-based protein quantitation method with a Direct Detect Infrared Spectrometer (Merck). Fifty or twenty-five (depending on the protein detection) micrograms of proteins was separated on 9% Tricine gels and transferred to polyvinylidene difluoride membranes (Merck Millipore). The membranes were incubated separately with anti-Foxn1 (1:1000, Cat#ab113235, Abcam), anti-p21 (1:1000, Cat# ab188244, Abcam), anti-Nrf-2 (1:1000, Cat#PA5-67,520, ThermoFisher), anti-Txnrd3 (1:1000, Cat# 19,517–1-AP,ThermoFisher), anti-HIF-1α (1:200, Cat# 16,066, Abcam), anti-Hif1an (Fih-1) (1:1000, Cat# ab187524,Abcam), or anti-β-actin (1:1000, Cat# 8226, Abcam), followed by incubation with fluorescent secondary antibodies against Alexa Fluor 594 (1:1000, Cat#A11037, Invitrogen) or Alexa Fluor Plus 800 (1:1000, Cat#A32789, Invitrogen) for 1 h. Bands were visualized using the ChemiDoc Touch Imaging System (Bio-Rad Laboratories, Inc) and analyzed using Image Lab Software (Bio-Rad Laboratories, Inc) according to the manufacturer’s protocol.

### RNA isolation and quantitative RT-PCR analysis

Frozen skin samples (*n* = 8 per group) collected from young (20-days-old), middle-age (1-year-old) and old (2-year-old) Foxn1 ^+/+^ and Foxn1^+/−^ mice were powdered in liquid nitrogen using a prechilled mortar and pestle, and homogenized in TRI Reagent. Total RNA was isolated from skin samples using TRI Reagent (Invitrogen, Thermo Fisher Scientific), according to the protocol provided by the manufacturer. RNA concentration and quality were evaluated spectrophotometrically with a NanoDrop 1000 system (Thermo Fisher Scientific) and verified by agarose gel electrophoresis. Complementary DNA (cDNA) was synthesized from 500 ng of total RNA using the High-Capacity cDNA Reverse Transcription Kit with RNase Inhibitor (Applied Biosystems, Thermo Fisher Scientific). For measurement of the mRNA levels of: Foxn1 (Cat# Mm01298129_m1), Collagen type 1 alpha 1 (Cat#Mm00801666_g1), Collagen type3alpha1(Cat#Mm01254476_m1), Elastin(Cat#Mm00514670_m1), p16/Cdkn2a(Cat#Mm00494449_m1), p21/Cdkn1a(Cat#Mm00432448_m1), Hif1α(Cat#Mm00468869), Fih1(Cat#Mm01198376_m1), Nrf2(Cat#Mm00477784_m1), Txn1(Cat#Mm00726847_s1), Txnrd3(Cat#Mm00462552_m1,Srxn1(Cat#Mm00769566_m1), Txn2(Cat#Mm00444931_m1), Txnrd1(Cat#Mm00443675_m1), Txnrd2(Cat#Mm00496766_m1) TaqMan Gene Expression Assays (Life Technologies, Thermo Fisher Scientific). were used. Based on prior validation, Hypoxanthine phosphoribosyltransferase 1 (Hprt1, Cat# Mm01545399_m1) was identified as the most stable reference gene for normalization in the context of the skin [24]. All samples were analyzed in duplicates. Each assay included a standard curve prepared from pooled skin aliquots. RNA amplification was carried out using the 7900HT Fast Real-Time PCR System under conditions: an initial denaturation at 95 °C for 10 min, followed by 40 cycles of 95 °C for 15 s and 60 °C for 1 min. mRNA expression levels were normalized to the reference gene Hprt1 and then multiplied by a factor of 10.

### Statistical analysis

Statistical analyses were performed with GraphPad Prism, Version 10.0.2 (GraphPad Software, La Jolla, CA). All data were assessed for normality using the Shapiro–Wilk test. Unpaired *t*-test normalized for multiple comparisons or two-way analysis of variance (ANOVA) with Tukey’s multiple comparisons was used. All data are expressed as the mean ± standard error (SEM). A value of *p* < 0.05 was considered statistically significant; *p < 0.05, **p < 0.01, ***p < 0.001 and ****p < 0.0001.

## Supplementary Information

Additional file 1: Fig. S1 (A) Foxn1 mRNA expression in skin of young (20-day-old), middle-age (1-year-old), and aged (2-year-old) Foxn1^+/+^ and Foxn1^+/−^ mice (*n* = 8 skin samples per group), (B,C) Foxn1, densitometric analysis and representative western blot with β-actin (reference protein) in the skin of young, middle-adult, and aged Foxn1^+/+^ and Foxn1^+/−^ mice (*n* = 3 skin samples per group). (D) Representative immunofluorescence images of Foxn1 (green) and DAPI (blue, nuclei) in skin sections from Foxn1⁺^/^⁺ and Foxn1^+/−^ mice at three age points: young (20 days), middle-age (1 year), and aged (2 years). Foxn1 shows nuclear localization within epidermal keratinocytes (E) and hair follicles (HF) in both genotypes across all age groups. Insets show magnified views of the boxed regions, highlighting epidermal Foxn1 localization. E, epidermis; D, dermis; HF, hair follicle. Scale bars: 20 µm (overview), 5 µm (insets). *n* = 1 mice per group. Fig. S2 The calculation of hair follicles (HF) numbers in dermis (A) and in dermal white adipose tissue (dWAT), (B) in the skin of young (20-days-old), middle-age (1-year-old) and aged (2-year-old) Foxn1^+/+^ and Foxn1^+/−^ mice. The bars indicate mean ± SEM ***p < 0.001, ****p < 0.0001. Fig. S3 Evaluation of transitional collagen fibers: orange (A, C, E) and yellow (B, D, F) in skin sections collected from young (20-days-old), middle-age (1-year-old) and aged (2-years-old) Foxn1^+/+^ and Foxn1^+/− ^mice (*n* = 3–4 per group). The bars indicate mean ± SEM *p < 0.05, **p < 0.01. Fig. S4 (A) Immunohistochemical detection of Txn1 in the skin of 1-year-old and 2-years-old Foxn1^+/+^ and Foxn1^+/−^ mice. mRNA expression of Txnrd1 (B), Txn2 (C) and Txnrd2 (D) determined by RT-qPCR analysis of skin tissues collected from 20-days-old, 1-year-old and 2-years-old Foxn1^+/+^ and Foxn1^+/−^ mice (*n* = 8 skin samples per group). (E) Immunofluorescent colocalization of Txnrd3 and Foxn1 (eGFP) detected on skin samples collected from 20-days-old transgenic Foxn1::Egfp mice. Dotted lines separate epidermis from dermis. e-epidermis; d-dermis; hf-hair follicles,. Scale bar (A)200 μm, (E)100 μm (confocal images; inset 20 μm). Fig. S5 (A) Hif-1α, (D) Fih-1, mRNA expression in skin of young (20-day-old), middle-age (1-year-old), and aged (2-years-old) Foxn1^+/+^ and Foxn1^+/−^ mice (*n* = 8 skin samples per group), (B,C) Hif-1α, (E,F) Fih-1, densitometric analysis and representative western blot with β-actin (reference protein) in the skin of young, middle-adult, and aged Foxn1^+/+^ and Foxn1^+/−^ mice (*n* = 3 skin samples per group).

Additional file 2: Fig. S1 Full western blots shown in the Additional File 1: Fig. S1C. Fig. S2 Full western blots shown in the Fig. 4D. Fig. S3 Full western blots shown in the Fig. 5E, H. Fig. S4 Full western blots shown in the Additional File 1: Fig. S5C, F.

## Data Availability

All data generated or analysed during this study are included in this published article and its supplementary information files.

## Abbreviations

BSA: Bovine serum albumin
Cdkn1a: Cyclin-dependent kinase inhibitor 1A (p21)
Cdkn2a: Cyclin-dependent kinase inhibitor 2A (p16)
DAPI: 4′,6-Diamidino-2-phenylindole
dWAT: Dermal white adipose tissue
ECM: Extracellular matrix
eGFP: Enhanced green fluorescent protein
EMT: Epithelial-mesenchymal transition
Fih-1 (Hif1an): Factor inhibiting HIF-1
Foxn1: Forkhead box N1
HF(s): Hair follicle(s)
HIF-1α: Hypoxia-inducible factor 1-alpha
Hprt1: Hypoxanthine phosphoribosyltransferase 1
IgG: Immunoglobulin G
K10: Keratin 10
K14: Keratin 14
LC–MS/MS: Liquid chromatography–tandem mass spectrometry
Lori: Loricrin
MMP(s): Matrix metalloproteinase(s)
NGS: Normal goat serum
Nrf2: Nuclear factor erythroid 2-related factor 2
PB: Phosphate buffer
PBS: Phosphate-buffered saline
PFA: Paraformaldehyde
PMSF: Phenylmethanesulfonyl fluoride
RIPA: Radioimmunoprecipitation assay (buffer)
ROS: Reactive oxygen species
TBS-T: Tris-buffered saline with Tween
TIMP(s): Tissue inhibitor(s) of metalloproteinases
Txn1/Txn2 (TXN): Thioredoxin 1/2
Txnrd1–3: Thioredoxin reductase 1–3
Srxn1: Sulfiredoxin 1
WGA: Wheat germ agglutinin

## References

[1] Diloreto R, Murphy CT. The cell biology of aging. Mol Biol Cell. 2017;26(25):4524–31. 10.1091/mbc.E14-06-1084 PMC4678010 26668170

[2] World Health Organization. Ageing. https://www.who.int/health-topics/ageing. Accessed 29 Jun 2026.

[3] Parrado C, Mercado-Saenz S, Perez-Davo A, Gilaberte Y, Gonzalez S, Juarranz A. Environmental stressors on skin aging. Mechanistic insights. Front Pharmacol. 2019;9(10):759. 10.3389/fphar.2019.00759 PMC6629960 31354480

[4] Cooper S. The biology of the skin. J R Soc Med. 2002;95(2):109. PMCID: PMC1279330.

[5] Gurtner GC, Werner S, Barrandon Y, Longaker MT. Wound repair and regeneration. Nature. 2008;453:314–21. 10.1038/nature07039 18480812

[6] Friedman O. Changes associated with the aging face. Facial Plast Surg Clin North Am. 2005;13(3):371–80. 10.1016/j.fsc.2005.04.004 16085282

[7] Hussein RS, Bin Dayel S, Abahussein O, El-Sherbiny AA. Influences on skin and intrinsic aging: biological, environmental, and therapeutic insights. J Cosmet Dermatol. 2025;24(2):e16688. 10.1111/jocd.16688 PMC11845971 39604792

[8] Bergfeld WF. The aging skin. Int J Fertil Womens Med. 1997;42(2):57–66. PMID: 9160214. 9160214

[9] Shin SH, Lee YH, Rho NK, Park KY. Skin aging from mechanisms to interventions: focusing on dermal aging. Front Physiol. 2023;14:1195272. 10.3389/fphys.2023.1195272 PMC10206231 37234413

[10] Quan T, Fisher GJ. Role of age-associated alterations of the dermal extracellular matrix microenvironment in human skin aging: a mini-review. Gerontology. 2015;61(5):427–34. 10.1159/000371708 PMC4524793 25660807

[11] Ortonne JP. Pigmentary changes of the aging skin. Br J Dermatol. 1990;122(Suppl 35):21–8. 10.1111/j.1365-2133.1990.tb16121.x 2186781

[12] Uitto J. Connective tissue biochemistry of the aging dermis. Age-associated alterations in collagen and elastin. Clin Geriatr Med. 1989;5(1):127–47. PMID: 2645992. 2645992

[13] Marcos-Garcés V, Molina Aguilar P, Bea Serrano C, García Bustos V, Benavent Seguí J, Ferrández Izquierdo A, et al. Age-related dermal collagen changes during development, maturation and ageing - a morphometric and comparative study. J Anat. 2014;225(1):98–108. 10.1111/joa.12186 PMC4089350 24754576

[14] Langton AK, Graham HK, Griffiths CEM, Watson REB. Ageing significantly impacts the biomechanical function and structural composition of skin. Exp Dermatol. 2019;28(8):981–4. 10.1111/exd.13980 PMC6851988 31152614

[15] Fisher GJ, Sachs DL, Voorhees JJ. Ageing: collagenase-mediated collagen fragmentation as a rejuvenation target. Br J Dermatol. 2014;171(3):446–9. 10.1111/bjd.13267 25234059

[16] Thomas DR. Age-related changes in wound healing. Drugs Aging. 2001;18(8):607–20. 10.2165/00002512-200118080-00005 11587247

[17] Birch-Machin MA, Bowman A. Oxidative stress and ageing. Br J Dermatol. 2016;175(Suppl 2):26–9. 10.1111/bjd.14906 27667312

[18] Yeo EJ. Hypoxia and aging. Exp Mol Med. 2019;51(6):1–15. 10.1038/s12276-019-0233-3 PMC6586788 31221957

[19] Martyts A, Sachs D, Hiebert P, Junker H, Robmann S, Hopf R, et al. Biomechanical and biochemical changes in murine skin during development and aging. Acta Biomater. 2024;186:316–29. 10.1016/j.actbio.2024.07.013 39009208

[20] Vu R, Jin S, Sun P, Haensel D, Nguyen QH, Dragan M, et al. Wound healing in aged skin exhibits systems-level alterations in cellular composition and cell-cell communication. Cell Rep. 2022;40(5):111155. 10.1016/j.celrep.2022.111155 PMC9901190 35926463

[21] Rezvani HR, Ali N, Serrano-Sanchez M, Dubus P, Varon C, Ged C, et al. Loss of epidermal hypoxia-inducible factor-1α accelerates epidermal aging and affects re-epithelialization in human and mouse. J Cell Sci. 2011;124(Pt 24):4172–83. 10.1242/jcs.082370 22193962

[22] Tohgasaki T, Nishizawa S, Yu X, Kondo S, Ishiwatari S. Thioredoxin promotes the regeneration and binding of elastic fibre and basement membrane. Int J Cosmet Sci. 2024;46(5):786–94. 10.1111/ics.12964 38685741

[23] Yang B, Lin Y, Huang Y, Shen YQ, Chen Q. Thioredoxin (Trx): a redox target and modulator of cellular senescence and aging-related diseases. Redox Biol. 2024;70:103032. 10.1016/j.redox.2024.103032 PMC10827563 38232457

[24] Kopcewicz MM, Kur-Piotrowska A, Bukowska J, Gimble JM, Gawronska-Kozak B. Foxn1 and Mmp-9 expression in intact skin and during excisional wound repair in young, middle-age, and old C57Bl/6 mice. Wound Repair Regen. 2017;25(2):248–59. 10.1111/wrr.12524 28371152

[25] Kur-Piotrowska A, Bukowska J, Kopcewicz MM, Dietrich M, Nynca J, Slowinska M, et al. Foxn1 expression in keratinocytes is stimulated by hypoxia: further evidence of its role in skin wound healing. Sci Rep. 2018;8(1):5425. 10.1038/s41598-018-23794-5 PMC5882803 29615703

[26] Walendzik K, Kopcewicz M, Bukowska J, Panasiewicz G, Szafranska B, Gawronska-Kozak B. The transcription factor FOXN1 regulates skin adipogenesis and affects susceptibility to diet-induced obesity. J Invest Dermatol. 2020;140(6):1166-1175.e9. 10.1016/j.jid.2019.11.010 31811821

[27] Machcinska S, Kopcewicz M, Bukowska J, Walendzik K, Gawronska-Kozak B. Impairment of the Hif-1α regulatory pathway in Foxn1-deficient (Foxn1-/-) mice affects the skin wound healing process. FASEB J. 2021;35(2):e21289. 10.1096/fj.202001907R PMC12315509 33475195

[28] Machcinska S, Walendzik K, Kopcewicz M, Wisniewska J, Rokka A, Pääkkönen M, et al. Hypoxia reveals a new function of Foxn1 in the keratinocyte antioxidant defense system. FASEB J. 2022;36(8):e22436. 10.1096/fj.202200249RR PMC12166283 35792861

[29] Prowse DM, Lee D, Weiner L, Jiang N, Magro CM, Baden HP, et al. Ectopic expression of the nude gene induces hyperproliferation and defects in differentiation: implications for the self-renewal of cutaneous epithelia. Dev Biol. 1999;212(1):54–67. 10.1006/dbio.1999.9328 10419685

[30] Lee D, Prowse DM, Brissette JL. Association between mouse nude gene expression and the initiation of epithelial terminal differentiation. Dev Biol. 1999;208(2):362–74. 10.1006/dbio.1999.9221 10191051

[31] Weiner L, Han R, Scicchitano BM, Li J, Hasegawa K, Grossi M, et al. Dedicated epithelial recipient cells determine pigmentation patterns. Cell. 2007;130(5):932–42. 10.1016/j.cell.2007.07.024 17803914

[32] Gawronska-Kozak B, Grabowska A, Kur-Piotrowska A, Kopcewicz M. Foxn1 transcription factor regulates wound healing of skin through promoting epithelial-mesenchymal transition. PLoS ONE. 2016;11(3):e0150635. 10.1371/journal.pone.0150635 PMC4777299 26938103

[33] Walendzik K, Kopcewicz M, Wiśniewska J, Opyd P, Machcińska-Zielińska S, Gawrońska-Kozak B. Dermal white adipose tissue development and metabolism: the role of transcription factor Foxn1. FASEB J. 2023;37(10):e23171. 10.1096/fj.202300873RR 37682531

[34] Gawronska-Kozak B. Scarless skin wound healing in FOXN1 deficient (nude) mice is associated with distinctive matrix metalloproteinase expression. Matrix Biol. 2011;30(4):290–300. 10.1016/j.matbio.2011.04.004 PMC3114183 21539913

[35] Terszowski G, Müller SM, Bleul CC, Blum C, Schirmbeck R, Reimann J, et al. Evidence for a functional second thymus in mice. Science. 2006;312(5771):284–7. 10.1126/science.1123497 16513945

[36] Junqueira LC, Bignolas G, Brentani RR. Picrosirius staining plus polarization microscopy, a specific method for collagen detection in tissue sections. Histochem J. 1979;11(4):447–55. 10.1007/BF01002772 91593

[37] Wolf MT, Carruthers CA, Dearth CL, Crapo PM, Huber A, Burnsed OA, et al. Polypropylene surgical mesh coated with extracellular matrix mitigates the host foreign body response. J Biomed Mater Res A. 2014;102(1):234–46. 10.1002/jbm.a.34671 PMC3808505 23873846

[38] Russell-Goldman E, Murphy GF. The pathobiology of skin aging: new insights into an old dilemma. Am J Pathol. 2020;190(7):1356–69. 10.1016/j.ajpath.2020.03.007 PMC7481755 32246919

[39] Varani J, Dame MK, Rittie L, Fligiel SE, Kang S, Fisher GJ, et al. Decreased collagen production in chronologically aged skin: roles of age-dependent alteration in fibroblast function and defective mechanical stimulation. Am J Pathol. 2006;168(6):1861–8. 10.2353/ajpath.2006.051302 PMC1606623 16723701

[40] Quan T, Qin Z, Robichaud P, Voorhees JJ, Fisher GJ. CCN1 contributes to skin connective tissue aging by inducing age-associated secretory phenotype in human skin dermal fibroblasts. J Cell Commun Signal. 2011;5(3):201–7. 10.1007/s12079-011-0144-0 PMC3145867 21720918

[41] Papaccio F, D’Arino A, Caputo S, Bellei B. Focus on the contribution of oxidative stress in skin aging. Antioxidants (Basel). 2022;11(6):1121. 10.3390/antiox11061121 PMC9220264 35740018

[42] Hansen JM, Zhang H, Jones DP. Differential oxidation of thioredoxin-1, thioredoxin-2, and glutathione by metal ions. Free Radic Biol Med. 2006;40(1):138–45. 10.1016/j.freeradbiomed.2005.09.023 16337887

[43] Oberacker T, Bajorat J, Ziola S, Schroeder A, Röth D, Kastl L, et al. Enhanced expression of thioredoxin-interacting-protein regulates oxidative DNA damage and aging. FEBS Lett. 2018;592(13):2297–307. 10.1002/1873-3468.13156 PMC6099297 29897613

[44] Sun L, Guo J, Brown R, Amagai T, Zhao Y, Su DM. Declining expression of a single epithelial cell-autonomous gene accelerates age-related thymic involution. Aging Cell. 2010;9(3):347–57. 10.1111/j.1474-9726.2010.00559.x PMC2894280 20156205

[45] Reis MD, Csomos K, Dias LP, Prodan Z, Szerafin T, Savino W, et al. Decline of FOXN1 gene expression in human thymus correlates with age: possible epigenetic regulation. Immun Ageing. 2015;12:18. 10.1186/s12979-015-0045-9 PMC4625732 26516334

[46] Thomas R, Wang W, Su DM. Contributions of age-related thymic involution to immunosenescence and inflammaging. Immun Ageing. 2020;17:2. 10.1186/s12979-020-0173-8 PMC6971920 31988649

[47] Liang Z, Dong X, Zhang Z, Zhang Q, Zhao Y. Age-related thymic involution: mechanisms and functional impact. Aging Cell. 2022;21(8):e13671. 10.1111/acel.13671 PMC9381902 35822239

[48] Mecklenburg L, Tychsen B, Paus R. Learning from nudity: lessons from the nude phenotype. Exp Dermatol. 2005;14(11):797–810. 10.1111/j.1600-0625.2005.00362.x 16232301

[49] Baker DJ, Childs BG, Durik M, Wijers ME, Sieben CJ, Zhong J, et al. Naturally occurring p16(Ink4a)-positive cells shorten healthy lifespan. Nature. 2016;530(7589):184–9. 10.1038/nature16932 PMC4845101 26840489

[50] Ogrodnik M, Carlos Acosta J, Adams PD, d’Adda di Fagagna F, Baker DJ, Bishop CL, et al. Guidelines for minimal information on cellular senescence experimentation in vivo. Cell. 2024;187(16):4150–75. 10.1016/j.cell.2024.05.059 PMC11790242 39121846

[51] Devgan V, Nguyen BC, Oh H, Dotto GP. P21WAF1/Cip1 suppresses keratinocyte differentiation independently of the cell cycle through transcriptional up-regulation of the IGF-I gene. J Biol Chem. 2006;281(41):30463–70. 10.1074/jbc.M604684200 16912042

[52] Freund A, Laberge RM, Demaria M, Campisi J. Lamin B1 loss is a senescence-associated biomarker. Mol Biol Cell. 2012;23(11):2066–75. 10.1091/mbc.E11-10-0884 PMC3364172 22496421

[53] Cheng L, Guo J, Sun L, Fu J, Barnes PF, Metzger D, et al. Postnatal tissue-specific disruption of transcription factor FoxN1 triggers acute thymic atrophy. J Biol Chem. 2010;285(8):5836–47. 10.1074/jbc.M109.072124 PMC2820809 19955175

[54] Zhang J, Xia B, Wakefield JS, Elias PM, Wang X. The role and implications of epidermal dysfunction in the pathogenesis of inflammaging. J Invest Dermatol. 2025;145(7):1580–8. 10.1016/j.jid.2024.09.025 39808093

[55] Valdivieso K, Rozmaric T, Victorelli S, et al. Transcription-independent induction of rapid-onset senescence is integral to healing. Nat Cell Biol. 2026;28:1281–99. 10.1038/s41556-026-01948-2 PMC13279267 42209725

[56] The Jackson Laboratory. When are mice considered old? 2017. https://www.jax.org/news-and-insights/jax-blog/2017/november/when-are-mice-considered-old.

[57] Kopcewicz M, Walendzik K, Bukowska J, Kur-Piotrowska A, Machcinska S, Gimble J, et al. Cutaneous wound healing in aged, high fat diet-induced obese female or male C57BL/6 mice. Aging (Albany NY). 2020;12:7066–111. 10.18632/aging.103064 PMC7202484 32294622

