# Supplementary material for: Foxn1 regulates epidermal structure, redox balance, and age related changes
Source: BMC Biol. 2026 Sep 16;24:216. doi: 10.1186/s12915-026-02737-x (PMC13660126; doi:10.1186/s12915-026-02737-x)
Supplement: Supplementary file 1 [file 12915_2026_2737_MOESM1_ESM.docx]

**Additional File 1: Fig. S1 (A)** *Foxn1* mRNA expression in skin of young (20-day-old), middle-age (1-year-old), and aged (2-years-old) Foxn1^+/+^ and Foxn1^+/-^ mice (n = 8 skin samples per group), **(B,C)** Foxn1, densitometric analysis and representative western blot with β-actin (reference protein) in the skin of young, middle-adult, and aged Foxn1^+/+^ and Foxn1 ^+/-^ mice (n = 3 skin samples per group). **(D)** Representative immunofluorescence images of Foxn1 (green) and DAPI (blue, nuclei) in skin sections from Foxn1⁺/⁺ and Foxn1⁺/⁻ mice at three age points: young (20 days), middle-age (1 year), and aged (2 years). Foxn1 shows nuclear localization within epidermal keratinocytes (E) and hair follicles (HF) in both genotypes across all age groups. Insets show magnified views of the boxed regions, highlighting epidermal Foxn1 localization. E, epidermis; D, dermis; HF, hair follicle. Scale bars: 20µm (overview), 5 µm (insets). n = 1 mice per group.

**D**

**Additional File 1: Fig. S2** The calculation of hair follicles (HF) numbers in dermis **(A)** and in dermal white adipose tissue (dWAT), **(B)** in the skin of young (20-days-old), middle-age (1-year-old) and aged (2-years-old) Foxn1^+/+^ and Foxn1^+/-^ mice. The bars indicate mean ±SEM ***p < 0.001, ****p < 0.0001.

**Additional File 1: Fig. S3** Evaluation of transitional collagen fibers: orange **(A, C, E)** and yellow **(B, D, F)** in skin sections collected from young (20-days-old), middle-age (1-year-old) and aged (2-years-old) Foxn1^+/+^ and Foxn1^+/-^ mice (*n*=3-4 per group). The bars indicate mean ±SEM *p < 0.05, **p < 0.01.

**Additional File 1: Fig. S4** **(A)** Immunohistochemical detection of Txn1 in the skin of 1-year-old and 2-years-old Foxn1^+/+^ and Foxn1^+/-^ mice. mRNA expression of *Txnrd1* **(B)***, Txn2* **(C)** *and Txnrd2* **(D)** determined by RT-qPCR analysis of skin tissues collected from 20-days-old, 1-year-old and 2-years-old Foxn1^+/+^ and Foxn1^+/-^ mice (*n* = 8 skin samples per group). **(E)** Immunofluorescent colocalization of Txnrd3 and Foxn1(eGFP) detected on skin samples collected from 20-days-old transgenic Foxn1::Egfp mice. Dotted lines separate epidermis from dermis. e-epidermis; d-dermis; hf-hair follicles,. Scale bar **(A)**200 μm, **(E)**100 μm (confocal images; inset 20 μm).

**Additional File 1: Fig. S5** **(A)** Hif-1α, **(D)** Fih-1, mRNA expression in skin of young (20-day-old), middle-age (1-year-old), and aged (2-years-old) Foxn1^+/+^ and Foxn1^+/-^ mice (n = 8 skin samples per group), **(B,C)** Hif-1α, **(E,F)** Fih-1, densitometric analysis and representative western blot with β-actin (reference protein) in the skin of young, middle-adult, and aged Foxn1^+/+^ and Foxn1 ^+/-^ mice (n = 3 skin samples per group).
