# Supplementary material for: Foxn1 regulates epidermal structure, redox balance, and age related changes
Source: BMC Biol. 2026 Sep 16;24:216. doi: 10.1186/s12915-026-02737-x (PMC13660126; doi:10.1186/s12915-026-02737-x)
Supplement: Supplementary file 2 [file 12915_2026_2737_MOESM2_ESM.docx]

**Additional File 2: Fig. S1** Full western blots shown in the Additional File 1: Fig. S1C

**Additional File 2: Fig. S2** Full western blots shown in the Fig. 4D.

**Additional File 2: Fig. S3** Full western blots shown in the Fig. 5E,H.

**Additional File 2: Fig. S4** Full western blots shown in the Additional File 1: Fig. S5C,F
